# It takes a community to hope: development and validation of the HOPE-4 scale in an Arab-Islamic context

**DOI:** 10.3389/fpsyg.2026.1798552

**Published:** 2026-04-20

**Authors:** Mohammed Mansouri, Timo Lorenz

**Affiliations:** 1 Department of Psychology and Educational Sciences, Djillali Liabes University of Sidi Bel Abbes, Sidi Bel Abbès, Algeria; 2 Department of Psychology, Medical School Berlin, Berlin, Germany

**Keywords:** Arab-Islamic context, cultural adaptation, hope, measurement invariance, positive psychology, scale validation

## Abstract

**Background:**

Hope research has been constrained by Western-centric models that prioritize individual agency and pathways, often neglecting relational and transcendent dimensions prominent in collectivist and religious societies.

**Method:**

Using a genetic algorithm, we developed and validated the HOPE-4 scale across two independent samples of Algerian adults (Study 1: *N* = 338; Study 2: *N* = 290; total *N* = 628), comprising four facets: Willpower (agency), Waypower (pathways), Faith-Based Hope (transcendent trust), and Communal Hope (relational solidarity).

**Results:**

Confirmatory factor analysis supported a hierarchical structure with excellent fit, high internal consistency (*α* = 0.93–0.95; *ω* = 0.96–0.97), strong convergent validity with established measures of hope and psychological capital, and moderate inter-facet correlations indicating discriminant validity. Based on approximate fit criteria, the scale showed configural, metric, and scalar invariance across gender and age groups. Scores also showed high consistency across demographic groups.

**Conclusion:**

These findings underscore the salience of faith-based and communal hope in Arab-Islamic contexts, extending traditional frameworks and addressing calls for emic instruments. The HOPE-4 provides a robust, culturally attuned tool for hope assessment, with implications for research and interventions in Arabic-speaking populations.

## Introduction

Hope is a cornerstone of positive psychology, associated with enhanced well-being, goal pursuit, adaptive coping, and resilience (Seligman and Csikszentmihalyi, 2000; Snyder, 2002). Meta-analyses indicate that hope-based interventions improve life satisfaction, alleviate depressive symptoms, and foster flourishing, yielding effects comparable to established therapies (Sin and Lyubomirsky, 2009). Snyder’s (2002) influential Hope Theory defines hope as a cognitive-motivational system comprising agency (motivational drive to pursue goals) and pathways (capacity to generate strategies amid obstacles), supporting progress in domains such as education, health, and performance (Gallagher et al., 2017; Snyder et al., 1991). Recent refinements, such as Leshem and Halperin’s (2024) bidimensional model, position hope at the intersection of desires (wishes for outcomes) and expectations (belief in achievability), integrating motivational and probabilistic elements particularly valuable in contexts of limited control.

### Cultural variations in hope

Despite these advances, hope research remains predominantly Western-centric, with over 80% of studies drawn from WEIRD (Western, Educated, Industrialized, Rich, Democratic) populations, limiting global applicability (Hendriks et al., 2019; Henrich et al., 2010). Dominant models like Snyder’s emphasize individual agency, control, and goal-directed cognition, reflecting autonomy and personal mastery central to individualistic cultures (Krafft et al., 2023a). Cross-cultural evidence reveals inconsistencies, such as variable factor structures for dispositional hope scales outside Western contexts (Bernardo and Ramos, 2024). In collectivist settings, hope often incorporates external sources such as family, peers, or spiritual beliefs with social and transcendent sources proving stronger predictors of well-being (Bernardo and Mendoza, 2021). These variations underscore that while hope may be universal, its expression, sources, and pathways are culturally embedded, necessitating “culturalized” theories that account for relational, normative, and spiritual dimensions (Bernardo and Ramos, 2024).

### Hope in the Arab-Islamic context

In Arab societies, hope (*Amal*) is profoundly shaped by Islamic theology and collectivist values. It represents not mere optimism but a spiritual virtue intertwined with *tawakkul* (trust in God’s plan), *sabr* (patient perseverance), and balanced moral action amid hardship (Al-Ghazali, as cited in Almulla and Abbasi, 2024; Osmani, 2008). Rooted in divine mercy affirmed in the Qur’an and Hadith, Arab hope often extends beyond individual goals to communal aspirations for family, *ummah* (community), and societal well-being (Basurrah et al., 2022; Bernardo and Ramos, 2024). Identity is relational, influenced by kinship, tribe, and shared faith, contrasting Western individualism (Markus and Kitayama, 1991; Realo, 2003). In adversity common amid political or economic challenges, hope draws strength from collective solidarity and transcendent trust rather than sole personal control (Leshem and Halperin, 2024; Mansouri and Lorenz, 2025). Thus, Arab hope is multifaceted, blending personal resilience with communal and spiritual dimensions divergent from Western frameworks.

### Measurement challenges and research gaps

Existing hope measures, such as Snyder’s Adult Hope Scale (AHS; Snyder et al., 1991) and the Herth Hope Index (HHI; Herth, 1992), demonstrate reliability in Western and some Arabic adaptations (Abdel-Khalek and Snyder, 2007; Alali, 2017; Fekih-Romdhane et al., 2025). However, systematic reviews reveal psychometric limitations, including infrequent assessment of content validity, responsiveness, or cross-cultural invariance (Redlich-Amirav et al., 2018). Arabic validations show promise but highlight gaps in capturing faith-based and collective facets (Alodat et al., 2024; Nasser, 2025). No emic framework fully integrates Arab-Islamic elements like tawakkul or communal solidarity, leaving underexplored how hope manifests in transitions, crises, or intergenerational transmission (Basurrah et al., 2022; Bernardo and Ramos, 2024).

### The present study

This study addresses these gaps by developing and validating the HOPE-4 framework, a culturally sensitive model comprising four facets: Willpower (agency), Waypower (pathways), Faith-Based Hope (transcendent trust), and Communal Hope (relational solidarity). Grounded in Snyder’s cognitive foundations while incorporating Arab-Islamic values of divine trust and collectivism, HOPE-4 aims to bridge Western individualism with non-WEIRD relational/spiritual dimensions. Two studies examine item optimization, confirmatory factor analysis, reliability, validity, and demographic invariance using independent Arabic samples.

### The proposed HOPE-4 framework

The HOPE-4 framework conceptualizes hope as a multidimensional construct comprising four interrelated facets: Willpower, Waypower, Faith-Based Hope, and Communal Hope. Willpower and Waypower capture cognitive-motivational aspects of hope, whereas Faith-Based Hope and Communal Hope capture transcendent and relational aspects that are especially salient in Arab-Islamic contexts. Thus, HOPE-4 measures hope as a multidimensional psychological resource that integrates universal cognitive-motivational processes with culturally embedded spiritual and relational dimensions. By assessing both self-directed and culturally embedded forms of hope, the HOPE-4 scale provides a contextually grounded operationalization of hope for Arab-Islamic settings.

Bernardo’s Locus of Hope model is an important reference point because it extends Snyder’s framework by distinguishing whether hope-related agency and pathways are attributed to the self or to external sources (e.g., family, peers, spiritual agents; Bernardo, 2010). HOPE-4 is related to this approach but addresses a different question. Rather than focusing on where agency and pathways are perceived to reside, HOPE-4 conceptualizes hope as a culturally grounded multidimensional construct and assesses four facets of hope as experienced in an Arab-Islamic context: Willpower, Waypower, Faith-Based Hope, and Communal Hope. In this framework, faith-based and communal dimensions are not only external supports for goal pursuit, but substantive forms of hope that include religious meaning-making, trust in divine timing, shared striving, and collective future orientation. Accordingly, HOPE-4 overlaps conceptually with locus-of-hope research while aiming to capture culturally specific content and organization of hope rather than source attribution alone.

### Willpower (agency hope)

Willpower is defined as the belief in one’s determination and motivational strength to pursue and sustain goal-directed actions despite obstacles, the “I can do this” component of hope. It encompasses inner drive, persistence, and self-confidence in personal competence, enabling individuals to initiate and maintain effort toward valued goals even amid uncertainty or setbacks (Snyder, 2002; Snyder et al., 1991). In dispositional hope theory, this corresponds to agency: the purposeful mental energy that fuels goal pursuit (Snyder, 2000).

While agency is well-established in Western frameworks, its expression may differ in contexts where systemic constraints limit personal control (Henrich et al., 2010; Leshem and Halperin, 2024). Cross-cultural research indicates that willpower remains resilient across cultures but frequently interacts with relational and spiritual resources, particularly in collectivist or high-adversity settings (Krafft et al., 2023a). For example, in South African and Nigerian samples, personal determination was sustained through communal support and spiritual trust rather than through individual mastery alone (Krafft et al., 2023c). Agency emerged as a significant predictor of perceived hope in several contexts, though its effects were weaker in relationally dominant samples where communal and spiritual resources complemented individual resolve (Krafft et al., 2023a).

In Arab-Islamic contexts, willpower is often bolstered by collective solidarity and trust in divine wisdom (Almulla and Abbasi, 2024). Personal determination does not operate in isolation but is scaffolded by family, community, and faith, a pattern consistent with interdependent self-construal (Markus and Kitayama, 1991). This cultural reality informs a deliberate design decision in the HOPE-4: rather than embedding relational and spiritual content within the Willpower items themselves, the present framework operationalizes Willpower as a cognitively distinct facet that captures the motivational core individuals experience as self-directed determination and persistence. The fact that this inner resolve is sustained by faith, family, and community in Arab-Islamic contexts is not ignored; it is systematically captured by the Faith-Based Hope and Communal Hope facets. This four-facet architecture allows the scale to represent both the universal cognitive-motivational substrate of hope and the culturally specific resources that sustain it, without conflating them at the item level. Accordingly, Willpower items are intentionally formulated to reflect agency as personally felt consistent with how self-directed motivation is phenomenologically experienced even in collectivist contexts, while the broader ecological scaffolding of that agency is assessed through the scale’s other dimensions.

Operationally, willpower is assessed through items that tap self-motivation, persistence, and reliance on inner strength (e.g., “I can motivate myself when things feel hard”; “My persistence helps me overcome obstacles”).

### Waypower (pathways hope)

Waypower is defined as the perceived ability to generate, identify, and flexibly adapt multiple strategies or routes to achieve goals, the “I will find a way” component of hope. It represents the cognitive and problem-solving dimension of hope, involving mental flexibility, creativity, and coping competence to navigate obstacles (Snyder, 2002; Snyder et al., 1991). In dispositional hope theory, this corresponds to pathways thinking: the cognitive capacity to envision viable routes even when initial paths are blocked (Snyder, 2000).

Although pathway thinking is conceptualized as an individual cognitive process in Western models, cross-cultural evidence suggests that pathways are often perceived as collectively or divinely shaped in non-WEIRD contexts (Bernardo and Ramos, 2024). Empirically, pathways thinking emerged as a consistent and often stronger predictor of perceived hope than agency across diverse samples (Krafft et al., 2023c). Openness to change values (self-direction, stimulation) positively predicted perceived hope in most samples, with cognitive activities (e.g., analyzing circumstances, information-seeking) showing context-dependent associations (Krafft et al., 2023a).

In trust-based analyses, cognitive-rational activities were confidence-driven in stable conditions but socially or spiritually scaffolded in uncertainty, supplemented by external factors like luck in low-religiosity contexts (Krafft et al., 2023a). Waypower thus appears relatively universal yet culturally modulated, prominent in individualistic settings through self-direction, but integrated with communal and transcendent resources in collectivistic or high-adversity contexts (e.g., South Africa, Nigeria).

For Arab societies, this integration is particularly salient. Flexible pathways may incorporate faith-based patience (sabr) and communal strategies, reflecting the cultural emphasis on collective problem-solving and trust in divine guidance (Almulla and Abbasi, 2024). This cross-cultural evidence shapes the conceptual positioning of Waypower in the HOPE-4, but with an important design clarification. Rather than incorporating relational or spiritual content into Waypower items directly, the present framework operationalizes Waypower as the cognitive-strategic dimension of hope, the individual’s perceived capacity to generate and flexibly adapt routes toward goals. This decision reflects the recognition that pathways thinking, even when culturally modulated, is phenomenologically experienced as a cognitive process: the person who breaks a goal into steps or adapts a plan is exercising strategic reasoning regardless of whether that capacity was cultivated through communal problem-solving or individual deliberation. The cultural and spiritual resources that shape or enable this cognitive capacity in Arab-Islamic contexts are captured structurally by the Faith-Based Hope and Communal Hope facets. In this way, the HOPE-4’s four-facet architecture is itself the solution to the challenge of cultural applicability: Willpower and Waypower assess the cognitive-motivational substrate of hope in experientially recognizable terms, while Faith-Based Hope and Communal Hope systematically capture the relational and transcendent dimensions that distinguish Arab-Islamic hope from its Western counterparts.

Operationally, waypower is assessed through items capturing strategic thinking, goal-planning, and adaptive problem-solving (e.g., “I can think of many ways to reach my goals”; “I can adapt my strategies for any situation”).

### Faith-based hope (religious/spiritual hope)

Faith-based hope is defined as trust that future outcomes are guided or supported by divine will, spiritual strength, or religious faith, providing meaning and resilience in uncertainty. It represents the transcendent dimension of hope, enabling endurance when personal agency and pathways are insufficient (Marcel, 1951; Pinsent, 2020). Theoretically, faith-based hope is framed as trust-based rather than confidence-based, emerging when personal control is limited and vulnerability requires openness to transcendent support (Earle and Siegrist, 2006; McGeer, 2008).

In Islamic thought, hope (Amal) is a spiritual virtue intertwined with tawakkul (trust in God’s plan) and sabr (patient perseverance), balancing aspiration with moral action and divine mercy [Al-Ghazali, as cited in Almulla and Abbasi (2024) and Osmani (2008)]. This conceptualization differs from purely cognitive models by emphasizing relational trust in a benevolent Higher Power as central to the hoping process.

Empirically, religious and spiritual sources of hope (e.g., experiencing divine support, answered prayers, prayer/meditation) are highly endorsed and strongly associated with perceived hope in religious contexts (Krafft et al., 2023c). Religiosity positively predicts perceived hope across samples, often integrating seamlessly with personal agency and prosocial motives (Krafft et al., 2023a). In high-hope contexts, faith and individual resolve operate synergistically rather than antagonistically, enhancing overall hopefulness (Krafft et al., 2023c). Faith-based hope is particularly salient in collectivist and religious societies, where it offers meaning and resilience when agency and pathways are constrained.

For the Arab-Islamic context, faith-based hope is essential to capture, as divine trust (tawakkul) intertwines with communal solidarity and meaning-making (Almulla and Abbasi, 2024). The HOPE-4 therefore includes this facet as a distinct dimension, reflecting the cultural reality that hope is not solely a product of personal cognition but also of transcendent relationship.

Operationally, faith-based hope is assessed through items capturing reliance on God, spiritual strength in hardship, and meaning-making through faith (e.g., “I believe God opens paths for me”; “Remembering God strengthens me in hardship”).

### Communal hope (collective hope)

Communal hope is defined as the belief that family, community, or society’s well-being can improve through shared effort, solidarity, and mutual support. It represents the relational and interdependent dimension of hope, involving collective visions, cooperative action, and trust in others’ care to realize common goods amid uncertainty (Braithwaite, 2004; McGeer, 2004).

Theoretically, communal hope extends beyond individualistic agency by emphasizing responsive hope rooted in mutual scaffolding individuals nurture one another’s agency through care, trust, and shared values (McGeer, 2004, 2008). In collectivist contexts, hope is not solely an internal state but emerges through relationships, reciprocity, and belonging (Erikson, 1959; Cobb and Green, 2017).

Empirically, social and prosocial sources of hope (e.g., family/friend support, helping others, contributing to meaningful causes) rank among the most highly endorsed and are strongly associated with perceived hope across cultures (Krafft et al., 2023c). Self-transcendence values benevolence (care for close others) and universalism (tolerance, justice, harmony) consistently predict perceived hope, with emotional support (both receiving and giving) proving more influential than instrumental support (Krafft et al., 2023a). Communal hope is particularly salient in collectivist or high-adversity societies (e.g., Latin American, African, Indian samples), where it enhances resilience without diminishing individual resolve (Krafft et al., 2023c).

In Arab societies, communal hope manifests as aspirations for collective well-being, intertwined with religious solidarity, kinship networks, and tribal bonds (Basurrah et al., 2022; Bernardo and Ramos, 2024). The concept of ummah (community of believers) extends hope beyond the individual to encompass family, community, and society. This relational orientation reflects interdependent self-construal, where identity and resilience emerge from connection rather than autonomy (Markus and Kitayama, 1991; Realo, 2003).

Operationally, communal hope is assessed through items capturing belief in collective efficacy, cooperation, and shared aspirations (e.g., “I believe cooperation leads to positive change”; “I want to build a better life for all of us”).

### Automated item selection

Recent methodological discussions have highlighted persistent concerns about measurement validity in psychological science (Flake et al., 2017; Hussey and Hughes, 2020). At the same time, concise questionnaires are often preferable when they can retain adequate psychometric quality (Fuchs and Diamantopoulos, 2009). Against this background, the present study uses automated item selection based on a genetic algorithm. Algorithmic approaches are increasingly used in psychological assessment and can provide transparent, criteria-based selection procedures that are less dependent on *ad hoc* manual decisions than traditional item reduction workflows (Algner and Lorenz, 2022; Lorenz, 2025; Kerber et al., 2022). When the optimization goal is defined *a priori* (e.g., model fit or reliability targets), such approaches can be especially effective and efficient (Leite et al., 2008; Olaru et al., 2015), and prior work suggests that resulting scales can perform similarly to, or sometimes better than, conventionally developed instruments (Sandy et al., 2017; Schroeders et al., 2016; Olaru and Danner, 2021). Importantly, automated selection is not a substitute for construct definition; the item pool still needs to be theory-guided and must cover the construct’s defining features (Dörendahl and Greiff, 2020).

Operationally, automated selection searches for an item subset from a larger pool that best approximates the intended measurement model, which turns scale construction into an optimization problem over many possible combinations (Schroeders et al., 2016; Kerber et al., 2022). Because enumerating all combinations is infeasible in practice, genetic algorithms use an iterative search strategy inspired by evolutionary principles (Holland, 1992; Schroeders et al., 2016). Such procedures are meta-heuristic and therefore do not guarantee the single best solution (Yarkoni, 2010), but they can substantially improve the psychometric profile of the final instrument while keeping computational effort manageable (Dörendahl and Greiff, 2020). A further advantage is that candidate solutions are evaluated at the level of complete item sets rather than single items; selection is typically guided by repeated CFAs so that item utility is judged in the context of the full model (Schultze, 2017).

## Methods

### Research design overview

This research employed a two-study, cross-sectional design to develop and validate the HOPE-4 scale, proceeding through three sequential stages: Study 1 (*N* = 338) focused on initial scale development, utilizing a genetic algorithm to select the optimal 16-item set from a 60-item pool and conducting a confirmatory factor analysis (CFA) to evaluate the hypothesized hierarchical structure and reliability; Study 2 (*N* = 290) served as an independent replication and validation phase, cross-validating the factor structure and establishing convergent and discriminant validity through correlations with the Adult Hope Scale (AHS) and the Revised Compound Psychological Capital Scale (CPC-12R); and a combined analysis (*N* = 628) was performed to test measurement invariance across gender and age groups to ensure demographic consistency.

### Participants

A total of 628 Arabic-speaking Algerian adults participated across both studies.

#### Study 1 (Initial development and validation sample)

This independent sample comprised 338 adults (primarily women, 83.4%; age range = 18–65 years, M = 32.29, SD = 9.51). The majority were single (76.3%), with balanced education levels (39.9% bachelor’s degree; 39.6% postgraduate). Approximately 61.2% were employed (47.3% in the public sector), with varied incomes (40.2% average; 24.3% above average; 23.1% below average). Regional distribution was balanced across Algeria’s four main areas: East (27.5%); North (26.3%); West (24.9%); South (21.0%).

#### Study 2 (Replication sample)

This sample included 290 adults (118 men, 40.7%; 172 women, 59.3%; age range = 18–75 years, M = 41.90, SD = 11.77). Most participants were married (66.2%), highly educated (72.4% held a postgraduate degree), employed (85.9%; predominantly public sector, 77.9%), and reported above-average income (61.0%). Regional distribution mirrored Study 1 (East: 27.2%; North: 26.6%; West: 22.1%; South: 23.8%).

### Procedure

The study was conducted between October 2025 and January 2026. Participants were recruited via convenience sampling through social media platforms, university networks, and community announcements. Inclusion criteria were Arabic-speaking Algerian adults aged 18 or older. No incentives were offered. In Study 1, participants completed the initial 60-item pool of the HOPE-4. In Study 2, participants completed the HOPE-4 along with the Adult Hope Scale and Revised Compound Psychological Capital Scale. Participants were fully informed about the study’s purpose, assured of confidentiality and anonymity, and encouraged to respond honestly. Participation was voluntary, and ethical standards adhered to national and institutional regulations and the 1964 Declaration of Helsinki and its amendments.

The study was preregistered on the Open Science Framework prior to data collection (OSF registration: https://osf.io/r8b6v). All sampling plans and analytic strategies were specified in the preregistration protocol.

### Measures

#### Hope scale (HOPE-4)

The development of the HOPE-4 began with an initial pool of 60 items, with 15 items drafted for each of the four proposed facets: Willpower, Waypower, Faith-Based Hope, and Communal Hope. The item pool was developed using a theory-driven and culturally grounded approach, drawing on (a) the hope literature (including cognitive-motivational models of hope), (b) cross-cultural work on relational and spiritual dimensions of hope, and (c) Arab-Islamic conceptualizations of hope (e.g., amal, tawakkul, sabr).

Items were initially written in parallel English and Algerian Arabic versions to ensure conceptual alignment across languages from the outset. Item writing was guided by the operational definitions of the four HOPE-4 facets specified in the introduction. The aim was to generate items that were (a) clear and concrete, (b) nonredundant within each facet, (c) representative of the facet’s content domain, and (d) culturally appropriate for Arabic-speaking adults.

To support content validity, the item pool was reviewed by an expert committee, who evaluated items for conceptual relevance, clarity, and cultural appropriateness. Revisions were made based on this review to improve semantic precision and reduce overlap across facets.

The item set then underwent a structured cross-cultural adaptation procedure following Beaton et al. (2000): forward translation by two bilingual experts (English to Algerian Arabic, prioritizing conceptual equivalence), back-translation by two independent bilingual experts, and committee-based reconciliation to ensure semantic, idiomatic, experiential, and conceptual equivalence. A pretest (*n* = 20) was subsequently conducted with Arabic-speaking adults to assess item clarity, comprehensibility, and cultural appropriateness. Minor wording refinements were made before the psychometric selection step.

All items were rated on a 6-point response scale (1 = strongly disagree to 6 = strongly agree). Higher scores indicate greater endorsement of the respective hope facet. The final 16-item HOPE-4 (4 items per facet) was selected from the 60-item pool using the preregistered automated item selection procedure described below.

#### Adult hope scale (AHS)

Dispositional hope was assessed using the Arabic version of the 12-item AHS (Alali, 2017). Four items assess agency, four assess pathways, and four are distractors (not scored). Items are rated on a 6-point rating scale from 1 = definitely false to 6 = definitely true; higher scores indicate greater hope. In this study, the scale achieved solid internal consistency (*α* = 0.95; *ω*t = 0.96).

#### Revised compound psychological capital scale (CPC-12R)

Psychological capital was assessed with the 12-item Arabic version of the CPC-12R (Mansouri and Lorenz, 2025), covering hope, efficacy, resilience, and optimism (3 items each). Items are rated on a 6-point rating scale from 1 = strongly disagree to 6 = strongly agree; higher scores indicate greater PsyCap. In this study, the scale achieved solid internal consistency (α = 0.93; ωt = 0.95).

### Data analysis

All analyses were conducted in R (Version 4.5.2; R Core Team, 2025). Data preparation, including recoding of variables (e.g., age grouped into three developmental stages: 18–30 years [emerging adults], 31–45 years [middle adults], and 46 + years [older adults]) and computation of facet scores (item means per facet), was performed using dplyr (Wickham et al., 2023). Descriptive statistics (means, standard deviations, skewness, kurtosis) for items and facets were calculated with psych (Revelle, 2024). Demographic characteristics were summarized using frequency tables and percentages in base R. Reliability was evaluated using Cronbach’s alpha (α) and McDonald’s omega (*ω*) in psych (Revelle, 2024).

For automated item selection, a genetic algorithm implemented in the R package stuart was applied to the initial pool of 60 items. The algorithm iteratively reduces the item pool by evaluating alternative item combinations with respect to their psychometric performance, operationalized via a fitness criterion reflecting overall item-set quality (Galán et al., 2013). Genetic algorithms rely on two fundamental mechanisms: variation and selection. Variation is introduced through recombination and mutation processes that generate novel item-set configurations, whereas selection prioritizes those configurations that best meet the predefined optimization criteria.

In this framework, individual items are treated as elemental units that are combined into candidate scales, conceptualized as chromosomes. An initial population of such chromosomes is randomly generated from the item pool, typically consisting of approximately 100 to 200 alternative item sets (Yarkoni, 2010). Across successive iterations, each generation of candidate scales is evaluated against the fitness function, and only the most psychometrically adequate solutions are retained for further refinement. During this process, genetic diversity is maintained through stochastic item exchanges within and between item sets, allowing the algorithm to explore a wide solution space. After a predefined number of iterations, the procedure converges on an item combination that demonstrates high psychometric robustness, yielding an efficient and well-performing scale solution (Schroeders et al., 2016).

The solutions were evaluated against an objective function consisting of a combination of the model fit criteria Root Mean Square Error of Approximation (RMSEA), Standardized Root Mean Square Residual (SRMR) and the Comparative Fit Index (CFI) as well as a composite reliability computed as McDonald’s ω.

Confirmatory factor analysis (CFA) for the HOPE-4 hierarchical model (four first-order facets loading onto a second-order general hope factor), as well as for the CPC-12R (Mansouri and Lorenz, 2025) and AHS (Alali, 2017), employed robust maximum likelihood (MLR) estimation in lavaan (Rosseel, 2012) to address non-normality. Model fit was assessed with robust CFI, TLI, RMSEA with 90% confidence interval, and SRMR. Average variance extracted (AVE) and composite reliability (CR) were derived from CFA models using semTools (Jorgensen et al., 2024). Measurement invariance across gender and age groups was tested via multi-group CFA with MLR estimation, evaluating configural, metric, and scalar invariance using changes in χ^2^ and approximate fit indices (ΔCFI, RMSEA, SRMR). Pearson correlations examined convergent and discriminant validity between HOPE-4 total/facet scores (item means) and validation measures (AHS total, agency, pathways; CPC-12R total) in Study 2. Group differences in HOPE-4 total and facet scores were tested in the combined sample (*N* = 628) using Welch’s independent-samples t-tests for binary variables and one-way analyses of variance (ANOVA) for multi-category variables, with Tukey HSD *post hoc* tests applied when ANOVA results were significant (base R; R Core Team, 2025). Effect sizes were reported as Cohen’s d (effsize package; Torchiano, 2020) and partial eta-squared (η^2^). All *p*-values are two-tailed (*p* < 0.05). Tables were formatted using knitr (Xie, 2025).

## Results

### Factor structure

The genetic algorithm selected 16 out of the original 60 items, with four items representing each of the four factors of hope. The final items are represented in Table 1. A hierarchical model with four first-order facets loading onto a second-order general hope factor was tested using CFA.

**Table 1:** English and Arabic wordings of the HOPE-4 Scale.

English version	Arabic version
**Willpower/agency**	**العزم والإصرار**
I keep moving toward my goals even when it takes a long time. I can motivate myself when things feel hard. I rely on my own inner strength. My persistence helps me overcome obstacles.	1. أواصل المضي نحو أهدافي حتى عندما يستغرق الأمر وقتًا طويلًا 2. أستطيع أن أحفّز نفسي عندما تكون الأمور صعبة 3. أعتمد على قوتي الداخلية 4. مثابرتي تساعدني على تجاوز العقبات
**Waypower/pathways**	الطرق والاستراتيجيات
I can think of many ways to reach my goals. I break big goals into smaller steps. I can map out clear steps toward my goals. I can adapt my strategies for any situation.	5. أستطيع أن أفكّر في عدة طرق للوصول إلى أهدافي 6. أقسم الأهداف الكبيرة إلى خطوات أصغر 7. أستطيع أن أحدد خطوات واضحة نحو أهدافي 8. أستطيع تكييف استراتيجياتي مع أي موقف
**Faith-based hope**	**الأمل القائم على الإيمان**
I believe God opens paths for me. Remembering God strengthens me in hardship. I believe outcomes arrive at the right time by God’s will. I believe God’s justice gives meaning to my struggles.	9. أؤمن أن الله يفتح لي طرقًا 10. تذكّر الله يقوّيني في الشدائد 11. أؤمن أن النتائج تأتي في الوقت المناسب بمشيئة الله 12. أؤمن أن عدل الله يمنح معنى لمعاناتي
**Communal hope**	**الأمل الجماعي المشترك**
I believe shared effort improves our future. I feel energized when I see people support each other. I believe cooperation leads to positive change. I want to build a better life for all of us.	13. أؤمن أن الجهد المشترك يحسّن مستقبلنا14. أشعر بالنشاط عندما أرى الناس يدعمون بعضهم بعضًا 15. أؤمن أن التعاون يقود إلى التغيير الإيجابي 16. أريد أن أبني حياة أفضل لنا جميعًا

In Study 1 (*N* = 338), the model HOPE-4 demonstrated excellent fit: χ^2^(100) = 130.177 (*p* = 0.023 standard; scaled *p* = 0.606); robust CFI = 1.000; robust TLI = 1.002; robust RMSEA = 0.000 (90% CI [0.000, 0.030]); SRMR = 0.039. All standardized loadings were statistically significant (*p* < 0.001), ranging from 0.683 (Item 16) to 0.961 (Item 9) on first-order factors and 0.786 (Communal Hope) to 0.902 (Waypower) on the second-order factor (Table 2).

**Table 2:** Standardized factor loadings for the 16-item HOPE-4.

Factor	Item	Study 1	Study 2
Willpower	Item 1	0.800	0.773
	Item 2	0.814	0.770
	Item 3	0.864	0.835
	Item 4	0.791	0.794
Waypower	Item 5	0.789	0.781
	Item 6	0.816	0.734
	Item 7	0.835	0.795
	Item 8	0.790	0.738
Faith-based hope	Item 9	0.961	0.935
	Item 10	0.898	0.871
	Item 11	0.939	0.875
	Item 12	0.904	0.917
Communal hope	Item 13	0.823	0.903
	Item 14	0.881	0.856
	Item 15	0.833	0.814
	Item 16	0.683	0.695
Second-order loadings	Willpower	0.873	0.887
	Waypower	0.902	0.769
	Faith-based hope	0.799	0.575
	Communal hope	0.786	0.616

All standardized loadings were statistically significant at *p* < 0.001.

In Study 2 (*N* = 290), the 16-item model exhibited acceptable to good fit: χ^2^(100) = 284.172, *p* < 0.001; robust CFI = 0.961; robust TLI = 0.954; robust RMSEA = 0.066 (90% CI [0.050, 0.081]); SRMR = 0.063. Standardized loadings were strong on first-order factors (range 0.695–0.935) and second-order factors (range 0.616–0.887).

### Descriptive statistics

In both samples, participants strongly endorsed hope items, with mean scores ranging from approximately 4.6 to 5.6 on the 6-point scale. Faith-Based Hope items received the highest means and exhibited the strongest negative skewness and positive kurtosis (leptokurtic distributions), indicative of ceiling effects and the profound cultural/religious salience of transcendent hope in the Algerian context. Willpower and Waypower items showed moderate means with milder skew and varied kurtosis, while Communal Hope items were intermediate. Standard deviations were adequate (typically 0.8–1.3), supporting good item discrimination. Facet-level descriptives (Table 3) revealed consistent patterns: Faith-Based Hope scored highest, followed by Communal Hope, Willpower, and Waypower. Distributions displayed moderate to strong negative skew particularly for Faith-Based Hope along with positive kurtosis in several facets, consistent with positive response tendencies in hope measures within collectivist and religious contexts. Skewness and kurtosis values were within acceptable ranges for parametric analyses (|skew| < 2, |kurtosis| < 7).

**Table 3:** Descriptive statistics for HOPE-4 facets.

	Min.	Max.	M	SD	Skewness	Kurtosis
Study 1 (*N* = 338)						
Willpower	1	6	4.89	0.88	−0.67	−0.01
Waypower	1	6	4.65	0.84	−0.26	−0.54
Faith-based	1	6	5.55	0.77	−1.48	0.64
Communal	1	6	5.08	0.86	−0.95	0.92
Study 2 (*N* = 290)						
Willpower	1	6	4.78	0.91	−0.72	0.67
Waypower	1	6	4.61	0.93	−0.71	0.56
Faith-based	1	6	5.43	0.93	−1.90	4.14
Communal	1	6	4.87	0.96	−0.94	1.42

M = Mean; SD = Standard Deviation.

### Reliability and measurement-level convergent validity

Internal consistency of the HOPE-4 scale was evaluated using Cronbach’s alpha (*α*) and McDonald’s omega (*ω*) for the total scale and each facet. In addition, AVE and CR were computed from the CFA models to assess measurement-level convergent validity. In Study 1 (*N* = 338), the total scale showed excellent reliability (α = 0.95, ω = 0.97). Facet reliabilities were: Willpower (α = 0.88, ω = 0.89), Waypower (α = 0.88, ω = 0.89), Faith-Based Hope (α = 0.98, ω = 0.98), and Communal Hope (α = 0.88, ω = 0.89). AVE values ranged from 0.65 to 0.91 (all ≥ 0.50), and CR ranged from 0.88 to 0.98, indicating high internal consistency and strong measurement-level convergent validity across samples.

In Study 2 (*N* = 290), the HOPE-4 total scale demonstrated excellent internal consistency (α = 0.93, ω = 0.96). Facet-level reliabilities were good to excellent: Willpower (α = 0.89, ω = 0.89), Waypower (α = 0.85, ω = 0.86), Communal Hope (α = 0.89, ω = 0.90), and Faith-Based Hope (α = 0.98, ω = 0.98). Convergent validity was supported by AVE values ranging from 0.58 (Waypower) to 0.92 (Faith-Based Hope); CR ranged from 0.85 to 0.97, all exceeding 0.70 (see Table 4).

**Table 4:** Reliability and measurement-level convergent validity indices for the HOPE-4.

	Measure/Facet	α	ω	AVE	CR
Study 1 (*N* = 338)	HOPE-4 total	0.95	0.97	-	-
	Willpower	0.88	0.89	0.65	0.88
	Waypower	0.88	0.89	0.65	0.88
	Faith-based hope	0.98	0.98	0.91	0.97
	Communal hope	0.88	0.89	0.65	0.88
Study 2 (*N* = 290)	HOPE-4 total	0.93	0.96	-	-
	Willpower	0.89	0.89	0.66	0.88
	Waypower	0.85	0.86	0.58	0.84
	Faith-based hope	0.98	0.98	0.92	0.97
	Communal hope	0.89	0.90	0.67	0.89

α = Cronbach’s alpha; ω = McDonald’s omega; AVE = average variance extracted; CR = composite reliability.

### External (nomological) and discriminant validity

External validity was examined in Study 2 through correlations with established measures of hope and psychological capital. Evidence for convergent validity with theoretically related constructs was observed, as the HOPE-4 total score correlated strongly with AHS agency (r = 0.73, *p* < 0.001), AHS pathways (r = 0.70, *p* < 0.001), and AHS total (r = 0.74, *p* < 0.001). The HOPE-4 total score also showed moderate to strong associations with CPC-12R total PsyCap (r = 0.65, *p* < 0.001), consistent with hope as a core component of psychological capital. At the facet level, Faith-Based Hope exhibited the strongest association with AHS total (r = 0.68, *p* < 0.001), followed by Willpower (r = 0.57, *p* < 0.001) and Waypower (r = 0.58, *p* < 0.001); Communal Hope showed a moderate correlation (r = 0.53, *p* < 0.001). All facets correlated positively with CPC-12R (r = 0.44–0.57, *p* < 0.001). Inter-facet correlations were moderate (r = 0.36–0.60), supporting discriminant validity among the four dimensions (Table 5).

**Table 5:** Correlations between the HOPE-4 facets, psychological capital, and adult hope scale.

	1	2	3	4	5	6	7	8	9
Willpower	-								
Waypower	0.60	-							
Faith-based hope	0.57	0.45	-						
Communal hope	0.50	36	0.59	-					
HOPE-4 total	0.84	0.76	0.81	0.77	-				
CPC-12R total	0.55	0.57	0.50	0.44	0.65	-			
AHS agency	0.59	0.56	0.67	0.51	0.73	0.69	-		
AHS pathways	0.52	0.56	0.65	0.51	0.70	0.69	0.88	-	
AHS total	0.57	0.58	0.68	0.53	0.74	0.71	0.97	0.97	-

CPC-12R = Revised Compound Psychological Capital Scale; AHS = Adult Hope Scale. All correlations are significant at *p* < 0.001 (two-tailed).

### Measurement invariance

Measurement invariance of the HOPE-4 scale was tested in the combined sample (*N* = 628) across gender (male vs. female) and age groups using multi-group confirmatory factor analysis (MG-CFA) with robust maximum likelihood estimation (MLR), and Satorra-Bentler scaled chi-square difference tests. Age was categorized into three theoretically meaningful developmental stages: 18–30 years (emerging adults), 31–45 years (middle adults), and 46 years and above (older adults), consistent with established life-span frameworks (Arnett, 2000). For gender, configural, metric, and scalar invariance were supported based on changes in approximate fit indices (ΔCFI ≤ 0.01), despite significant Satorra–Bentler scaled chi-square difference tests (all *p* < 0.001), which is common in larger samples.

For age groups, configural, metric, and scalar invariance were supported based on changes in approximate fit indices (ΔCFI ≤ 0.01), despite significant chi-square difference tests, indicating comparable factor structure, loadings, and intercepts across age groups (Table 6). Absolute fit indices (RMSEA ≤ 0.061; SRMR ≤ 0.044) were acceptable across models.

**Table 6:** Fit indices for measurement invariance across gender and age groups (*N* = 628).

	χ^2^	df	χ^2^/df	CFI	RMSEA (90% CI)	SRMR	∆CFI
Gender invariance							
Configural	333.74	196	1.70	0.985	0.047 (0.038–0.056)	0.028	-
Metric	344.17	208	1.65	0.985	0.046 (0.037–0.054)	0.031	0.000
Scalar	367.24	220	1.66	0.984	0.046 (0.038–0.054)	0.032	−0.001
Age groups							
Configural	512.10	294	1.74	0.977	0.060 (0.051–0.068)	0.034	-
Metric	546.20	318	1.71	0.976	0.059 (0.050–0.067)	0.041	−0.001
Scalar	611.58	342	1.78	0.972	0.061 (0.053–0.069)	0.044	−0.004

χ^2^ = Chi-square; df = degree of freedom; CFI = Comparative Fit Index; RMSEA = Root Mean Square Error of Approximation; SRMR = Standardized Root Mean Square Residual.

### Group differences

Group differences in HOPE-4 total and facet scores were examined in the combined sample (*N* = 628) using Welch’s independent-samples t-tests (for binary variables) and one-way ANOVA (for multi-category variables), with *post hoc* Tukey HSD tests applied when ANOVA results were statistically significant. Effect sizes were reported as Cohen’s d (for t-tests) and partial eta-squared (η^2^; for ANOVAs). No statistically significant differences emerged in HOPE-4 total scores across gender (t(344.51) = −0.37, *p* = 0.713, d = 0.03), age groups (18–30, 31–45, 46 + years; F(2, 625) = 0.67, *p* = 0.514, η^2^ = 0.002), region of residence (F(3, 622) = 1.02, *p* = 0.382, η^2^ = 0.005), employment status (F(2, 625) = 1.01, *p* = 0.364, η^2^ = 0.003), marital status (F(3, 624) = 1.81, *p* = 0.144, η^2^ = 0.009), educational level (F(5, 622) = 1.46, *p* = 0.199, η^2^ = 0.012), or income level (F(3, 624) = 2.18, *p* = 0.089, η^2^ = 0.010). Effect sizes were consistently small (d < 0.20; η^2^ < 0.02) across all comparisons.

Similar statistically non-significant patterns were observed for all four HOPE-4 facets (all *p* > 0.05; effect sizes small). These results indicate that HOPE-4 scores are relatively consistent across major demographic groups in the Algerian sample, supporting the scale’s robustness across the demographic groups examined in this sample.

## Discussion

The present study developed and validated the HOPE-4 scale, a multidimensional measure of hope comprising four facets: Willpower (agency), Waypower (pathways), Faith-Based Hope (transcendent trust), and Communal Hope (relational solidarity). Findings from two independent Algerian samples confirmed a hierarchical structure with robust fit, strong internal consistency, convergent validity with established measures, and discriminant validity among facets. The scale showed configural, metric, and scalar invariance across gender and age groups based on approximate fit criteria, despite significant chi-square difference tests, with scores showing consistency across demographic variables. These results extend cognitive-motivational models of hope, which center on individual agency and pathways (Snyder, 2002), by highlighting relational and transcendent dimensions prominent in collectivist and religious contexts (Bernardo and Ramos, 2024). The elevated endorsement of Faith-Based Hope aligns with Islamic views of hope (Amal) as a spiritual virtue rooted in tawakkul (reliance on divine wisdom) and sabr (patient endurance), balanced with righteous action and divine mercy (Al-Ghazali, as cited in Almulla and Abbasi, 2024; Osmani, 2008). This facet’s salience echoes cross-cultural patterns where transcendent trust sustains hope amid uncertainty, particularly in religious societies (Krafft et al., 2023c). Similarly, Communal Hope’s strength reflects interdependent self-construal in Arab cultures, where resilience emerges from kinship, ummah (community), and shared aspirations rather than autonomous pursuit (Markus and Kitayama, 1991; Realo, 2003; Basurrah et al., 2022). These patterns contrast with Western frameworks that prioritize personal mastery, often overlooking external loci such as family, peers, or spiritual beliefs that prove more predictive of well-being in non-WEIRD settings (Bernardo and Mendoza, 2021; Henrich et al., 2010). By integrating Snyder’s foundational elements with Arab-Islamic emphases on divine guidance and collective solidarity, HOPE-4 addresses key gaps in the literature: the Western-centric bias of dominant models (Hendriks et al., 2019), inconsistent cross-cultural replication of individual-focused scales (Bernardo and Ramos, 2024), and the underrepresentation of faith-based and communal sources in hope assessment (Krafft et al., 2023a; Redlich-Amirav et al., 2018). It responds to calls for emic, culturally embedded measures that capture hope’s distributed nature in relational and spiritual contexts (Bernardo and Ramos, 2024).

### Limitations

Several limitations should be considered when interpreting the present findings. First, the use of convenience sampling and online administration may limit representativeness. Participants were recruited via social media, university networks, and community announcements, which may have led to underrepresentation of rural populations, older adults, and individuals with lower digital access. In addition, the two samples differed in composition (e.g., Study 1 was predominantly female, whereas Study 2 was more balanced but highly educated and largely employed in the public sector), which may have influenced response patterns. These characteristics limit the strength of generalizations beyond the present samples.

Second, the exclusive focus on Algerian adults limits generalizability to the broader Arab world. Although the HOPE-4 was developed to reflect Arab-Islamic conceptions of hope, Arab societies differ in religiosity, socioeconomic conditions, political contexts, and forms of collectivism. The current findings, therefore, support the scale’s validity in an Algerian context but should not be assumed to transfer directly to other Arab populations without further cross-national validation.

Third, although convergent validity was supported through associations with the AHS and CPC-12R, the present validation relied primarily on self-report correlations and did not include broader external criteria (e.g., behavioral, longitudinal, or clinically relevant outcomes). In addition, discriminant validity evidence remains preliminary. Moderate inter-facet correlations support differentiation among the four HOPE-4 dimensions, but stronger tests (e.g., alternative structural models, external discriminant measures, and incremental validity analyses) are still needed. Future work should therefore expand the nomological network of HOPE-4 and evaluate whether the four facets show distinct predictive utility. We also did not include the Locus-of-Hope-Scale (Bernardo, 2010) in the present validation, so future studies should examine the empirical overlap and incremental validity of HOPE-4 relative to internal and external locus-of-hope dimensions.

Fourth, the cross-sectional design precludes conclusions about temporal stability, sensitivity to change, and predictive validity. The present study establishes internal structure and initial validity evidence, but it does not show whether HOPE-4 scores remain stable over time or predict later outcomes such as coping, well-being, or resilience in adverse contexts. Longitudinal and intervention-based studies are needed to assess test–retest reliability, responsiveness, and prognostic value.

A further limitation concerns the interpretation of the Faith-Based Hope facet. Because several items explicitly refer to God, divine will, and religious meaning-making, this facet may partially overlap with religiosity or religious coping, and the present study did not include an independent measure of religiosity to test discriminant validity directly. This also limits the facet’s applicability to less religious individuals and to religiously diverse populations, and the current wording should therefore be understood as culturally specific rather than universally generalizable. The very high factor loadings and internal consistency of this facet may indicate strong construct coherence, but they may also reflect a highly homogeneous religious content domain. Accordingly, the current findings support the salience of faith-based hope in this context, but they do not yet fully establish the empirical boundary between faith-based hope, religiosity, and religious coping.

Relatedly, some HOPE-4 items, particularly those assessing faith-based and communal hope, may be susceptible to social desirability bias because they reflect socially and morally valued orientations in the studied cultural context. This may have contributed to elevated endorsement levels and the ceiling tendencies observed for Faith-Based Hope, which in turn may reduce variance and constrain discrimination at the upper end of the scale. Ceiling effects can inflate internal consistency while limiting sensitivity to individual differences and potentially attenuating associations with external variables. At the same time, self-report remains an appropriate method for assessing subjective psychological constructs such as hope, provided that potential response biases are acknowledged and interpreted cautiously (Chan, 2010). Future studies should therefore include independent measures of religiosity/religious coping, social desirability (or other response-style indicators), and, where possible, analyses of differential item functioning to test whether item parameters vary across demographic groups. This would strengthen evidence for construct validity and help determine whether some items function differently across subpopulations.

Overall, these limitations do not negate the present contribution, but they indicate that the HOPE-4 should be viewed as an initial, culturally grounded validation with strong internal evidence and promising external validity, requiring further refinement and cross-context replication.

### Implications and future directions

Theoretically, HOPE-4 advances a pluralistic understanding of hope, illustrating how transcendent and communal elements scaffold cognitive processes in religious/collectivist societies (McGeer, 2008; Krafft et al., 2023b). Practically, it provides a culturally attuned tool for assessing hope in Arabic-speaking populations, with potential applications in clinical interventions, educational programs, and community initiatives that leverage faith and relational resources.

Future research should prioritize cross-national validation across diverse Arab contexts to examine regional nuances. Longitudinal and mixed-methods studies could explore HOPE-4’s predictive utility for mental health outcomes amid adversity, as well as intergenerational transmission through family and religious networks. Intervention trials testing facet-targeted strategies such as group-based activities enhancing Communal Hope or faith-integrated practices bolstering Faith-Based Hope would illuminate pathways to cultivate resilience. Ultimately, integrating HOPE-4 into broader positive psychology efforts could inform culturally sensitive policies addressing socio-economic and political challenges in the region. The HOPE-4 scale reveals hope as an intrinsically relational and spiritual resource in Arab societies, where Amal embodies patient trust in divine mercy and communal solidarity. By transcending individualistic paradigms, it affirms hope’s role as a profound source of endurance and meaning, paving the way for a more inclusive global positive psychology.

## Conclusion

The HOPE-4 scale offers a culturally attuned measure of hope that bridges universal cognitive-motivational processes with the transcendent and communal dimensions central to Arab-Islamic conceptions of Amal. By validating a hierarchical structure that integrates agency, pathways, faith-based trust, and relational solidarity, this study contributes a contextually relevant tool for assessing hope in Arabic-speaking populations, pending further validation in more diverse and representative samples across Arab contexts. The results underscore the importance of moving beyond Western-centric frameworks to capture hope as a multifaceted resource deeply embedded in spiritual reliance and collective resilience. This advancement supports more inclusive positive psychology research and practice, with potential to inform interventions that leverage faith and community in fostering well-being amid adversity. Future efforts should build on this foundation to refine and extend the scale across diverse Arab-Islamic contexts.

## Data Availability

All datasets, the complete R analysis script, and accompanying documentation are openly available in the Open Science Framework repository. No identifiable information is included in the shared data. Materials can be accessed at: https://doi.org/10.17605/OSF.IO/PHMDV.
